# “You’re damned if you do, you’re damned if you don’t”: a qualitative exploration of parent motives for provision of mobile screen devices in early childhood

**DOI:** 10.1186/s12889-022-14459-0

**Published:** 2022-11-02

**Authors:** Sumudu R. Mallawaarachchi, Merrilyn Hooley, Wendy Sutherland-Smith, Sharon Horwood

**Affiliations:** 1 School of Psychology, Deakin University, Burwood, Australia; 2 Centre for Research in Assessment and Digital Learning (CRADLE), Deakin University, Burwood, Australia

**Keywords:** Mobile device use, Mobile screen media, Early childhood, Parenting, Screen time

## Abstract

**Background:**

Exploring parental motives for providing smartphones and tablets to young children is important to better understand ways to optimise healthy use of mobile screens in early childhood. To date, no study has qualitatively examined the factors underpinning parental motives of providing mobile screens to young children, using a theoretically driven approach.

**Methods:**

We conducted 45 in-depth, semi structured online interviews with primary caregivers of toddlers and pre-schoolers from diverse family backgrounds who participated in a large online survey in Australia. Themes were generated from the transcribed interviews using template thematic analysis. The coding was completed deductively using the Theory of Planned Behaviour (TPB) and data-driven induction.

**Results:**

Participants consistently reported a spectrum of attitudes, subjective norms and perceived behavioural control aspects which drove their decision to provide or not provide a mobile screen device to their child. Five main descriptive themes were generated, guided by the TPB: (1) Convenience, connection, and non-traditional learning experience; (2) Negative behavioural consequences and potential activity displacement through mobile screens; (3) Influences of society and resources; (4) Managing and achieving a balance; (5) External challenges.

**Conclusions:**

Overall, the findings demonstrated that parents experienced cognitive dissonance between their attitudes and behaviour, primarily from perceived behavioural control and subjective norms negating the influence of attitudes on their motives to provide a device. These insights offer important avenues for public health messaging and resources to better involve and support parents in decision-making relating to mobile screens in everyday lives of young children.

**Supplementary Information:**

The online version contains supplementary material available at 10.1186/s12889-022-14459-0.

## Background

The increasing usage rates of mobile screen devices (i.e., smartphones and tablets) among toddlers and pre-schoolers [1–3] evidences the increasingly pervasive nature of technology use in early childhood. The potential for prolonged screen time by young children has been exacerbated by the ongoing COVID-19 pandemic [4–6] and pandemic-related changes such as online learning, working from home, and limited in-person social interaction opportunities. Nevertheless, there is limited qualitative research investigating early childhood mobile screen use and the implications for effective device management in the home. The portability, internet connectivity, unlimited curated content and multi-functionality of mobile devices are appealing features for young users, and tend to promote more solitary than shared use [7]. Given that parents or caregivers are typically the gatekeepers of technology access for toddlers and pre-schoolers, a major step towards better understanding the potential risks and benefits of smartphones and tablets in early childhood is to empirically study the beliefs, reasons and motivations for parents’ provision of mobile screen devices to their children [8].

### The role of parents in smartphone and tablet use in early childhood

The Interactional Theory of Child Problematic Media Use [9] is a theoretical representation of the ways in which excessive or prolonged screen media use in early childhood may interfere with typical early childhood functioning. Of the myriad factors (for example, child behaviour, household dynamics, parent–child relationship) associated with early childhood screen use, parents are thought to exert several salient proximal influences. First, parents are the main providers of smartphones and tablets to young children in household settings [10]. Second, parents’ own use can serve as a calibration point for what they may perceive as ‘normal’ use within society and for their children [9]. Third, parents’ own device usage habits and patterns will be modelled by their children through observational learning, normalizing their use for recreation and other purposes [11]. Fourth, when parental device engagement is high, it may reduce sensitivity to, or awareness of, their child’s needs. This is thought to be because parental screen absorption can potentially limit the effective interpretation of, and contingent responding to, their child’s cues for attention [12]. Finally, broader parenting attitudes about family socialization and boundary-setting may influence parents’ decisions about whether or not to provide mobile screens and how to regulate their use within the household [13].

Previous research has explored the relationship between parenting factors and traditional screen time (e.g., television and gaming consoles) in early childhood (see Xu et al., 2015 [14] for a review) and more recently contemporary screen media such as smartphones and tablets (e.g., [15, 16]). Factors tested include parenting practices [17, 18], parental perceptions of screen media use [19–21], parenting styles [22, 23] and parental self-efficacy in managing screen time [24, 25]. This research has largely undertaken a quantitative approach [15, 16, 26, 27], while qualitative studies have been relatively scarce [28–30]. Qualitative methodologies offer depth and richness in understanding diverse parent experiences, beliefs and behaviours [31]. Qualitative studies also complement quantitative findings and inform new directions in the area [32]. To date, qualitative studies have employed either ‘small Q’ or ‘thin’ data through qualitative surveys [29] or have been data-driven to generate themes [28, 30]. To our knowledge, no study has used a structured hybrid (deductive and inductive) descriptive approach to gain an in-depth understanding of factors that underpin parent decision-making and practices around early childhood use of mobile screens.

### Theory of planned behaviour

The present study employed a structured theoretical approach to understanding parent decision-making and practices as they pertain to the study aims. The Theory of Planned Behaviour (TPB; [33]) is a well-established model in health research that describes the attitudes, normative beliefs and perceived behavioural control that drive intentions and behaviour (see Hamilton et al. 2020 [34] for a review). The three main components of the TPB are; (1) Attitudes: the appraisal or evaluation of the possible outcomes of a particular behaviour; (2) Subjective norms: the perceived social pressure (i.e., expectations from other people) to engage or not engage in the behaviour; (3) Perceived behavioural control: the level of confidence in one’s capacity to engage or not engage in the behaviour [33]. Previously, the TPB has been a useful quantitative framework for understanding parents’ beliefs and decisions, and successfully predicted follow-up screen-related behaviours in early childhood [35, 36]. However, to date, no study has used the TPB to qualitatively assess parent decision-making and motives with respect to use of mobile screens in early childhood.

### The current study

Early childhood is an optimal period for implementing early interventions to manage the potential formation of problematic screen-use habits [37]. Habits formed in early childhood lay a foundation for later development of lifestyle patterns including diet, physical activity, and screen use [38]. Before recommending appropriate and nuanced actionable targets for early awareness and interventions, it is necessary to first understand parents’ attitudes towards, and motives for, providing mobile screen devices in early childhood. Accordingly, the current study aimed to explore the factors underpinning parents’ attitudes, subjective norms and perceived behavioural control with respect to toddler and pre-schooler smartphone or tablet use through the lens of the TPB.

## Methods

### Study design

The present qualitative study was part of an online longitudinal survey of young children’s smartphone and tablet use in Australia and was conducted from June to August 2021. Parents who took part in this survey were invited to participate in an online interview to discuss their views and experiences of smartphone and tablet use in early childhood. The study received ethics approval from the authors’ Institutional Human Research Ethics Committee (HEAG_H_234_2020).

This study assumed a limited realism philosophical position with a critical realist approach in viewing the research and its data. This position holds that ‘reality is out there’ and that through science, our knowledge will eventually approximate that reality. It also acknowledges that researchers’ quest for knowledge is limited in time and space and bound by current theory, evidence and methods of evaluation, and therefore is characterised by a-priori themes [39]. We adopted a constructivist epistemological stance, as we understand knowledge is constructed within different settings, times and situations. We therefore aimed to develop an account of participants’ viewpoints and experiences that is trustworthy and potentially transferable, while acknowledging conclusions drawn are tentative [40]. Quality checks through collaboration to stimulate critical thinking and fresh perspectives, maintaining audit trails using annotated coding templates and practicing reflexivity by acknowledging researcher subjectivity are encouraged in this approach [40], and were employed by our team.

In relation to reflexivity, the contribution of each researcher’s position and personal experience to the interpretation of the data were considered [41]. Our team are Australian residents, with two parents (WS-S and MH) and two non-parents (SM and SH), allowing both type of perspectives to be considered during analysis. The expertise of the team varied across human–computer interaction (SM and SH), child development (MH) and educational research methodologies (WS-S) to ensure the interpretation of data is conducted across multiple lenses and professional beliefs.

It is important to also acknowledge that the data collection took place during the second year of the COVID-19 pandemic. Our participants were from various states across Australia and exposed to varying lockdown restrictions and circumstances. We recognize that the stay-at-home directions and limited opportunities for travel and social interactions potentially influenced families in their decisions related to screen time and may have amplified their emotions and concerns related to parenting [42].

### Participants and recruitment

Eligible participants were English-speaking primary caregivers (parent or guardian) of a toddler or a pre-schooler aged one to six years living in Australia. Parents were recruited to the longitudinal survey via social media advertisements (e.g., Facebook, Reddit), through media exposure (e.g., television, newspaper) and snowballing. Of the 52 participants that expressed their interest in participating in the interviews, 45 were included in the final sample using a purposive sampling matrix (see Additional File 1). Seven of the participants who expressed their interest were subsequently unavailable or not contactable. Participants who volunteered were predominantly female, however, we ensured that all males (*N* = 2) who expressed interest were included in the final sample. The purposive sampling matrix helped to ensure diversity in characteristics and backgrounds given the important role of parent demographic factors, parenting styles, and associated attitudes in device use by young children [13]. Participation in the online interview was voluntary and did not affect participation in the overall study. All 45 participants were offered entry into a prize draw to win one of thirty $30 gift vouchers.

### Study procedure

The online interviews were conducted in English and hosted and recorded via the Zoom™ online platform. The interview followed a semi-structured format with primary (main) open-ended questions asked in each interview. A set of secondary questions (prompts) for each primary question served as a guide for clarification if needed. This format allows the interviewer to be guided by topics while also permitting flexibility in conversation [40]. Each interview lasted between 17 to 76 min with an average duration of 37 min. Six researchers who were trained on the standardized interview protocol conducted the interviews. After pilot testing, the team members determined that two interview schedules (see Additional File 2) were necessary. This was due to parents’ and caregivers’ eagerness to discuss their views at length on wide-ranging topics of inquiry which often resulted in excessively long interview durations. Hence, Schedules A and B were developed to overlap in some key areas of screen use. The two schedules branched out on topics that were important to parents and the research aims but that could not feasibly be included in a single interview. For example, both schedules comprised core research questions such as parental perceptions of early childhood screen use, but also had distinct aspects of interest such as perceptions on provision of devices (Schedule A) or perceptions on managing young children’s screen use (Schedule B). Prior to the interviews, participants were purposefully allocated to schedule A or B, matched on their demographic characteristics.

### Analysis

**Fig. 1:**
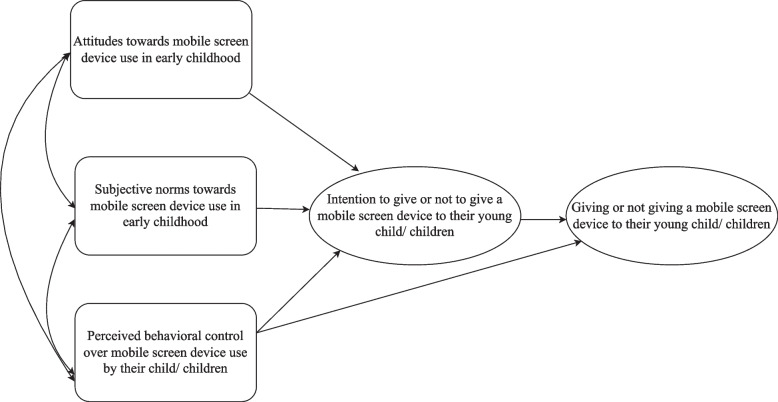
A-priori Themes adapted from the Theory of Planned Behaviour (Ajzen, 1991)

The qualitative interviews were recorded, transcribed, and imported to NVivo V.12 for coding. Template thematic analysis [43] was used to examine and identify the shared patterns and meanings across the data. This ‘codebook’ type of thematic analysis encourages efficiency in analysis [44, 45] and aims to maintain a balance between structure and flexibility in coding [46]. Hybrid inductive and deductive coding were used in the present study. A-priori themes (deductive coding) provided structure and guidance very early in the analysis as a lens through which to view the data, while also allowing the data to guide the identification of shared patterns and meanings within the themes (inductive data-driven coding). The a-priori themes also helped to focus on the areas of greater relevance to our research question and to avoid potentially redundant or repetitious coding [45]. A-priori themes were deemed appropriate for this study given the large number of interviews conducted by multiple researchers [45]. Themes were developed (illustrated in Fig. 1) using the components from the well-established Theory of Planned Behaviour (TPB; [33]).

The following series of steps were undertaken to complete the template analysis of the transcribed interviews: (1) familiarization with the data (SM, SH); (2) preliminary coding of initial sub-set of 16 interviews (SM); (3) clustering and organizing of initial themes – this involved modifying the a-priori themes and generating additional sub-themes that arose within codes (SM, SH); (4) producing the initial coding framework through collapsing of codes (SM, SH, in consultation with WS-S); (5) coding of the remaining 29 interviews (in three sub-sets of 10, 10 and 9) applying the coding template through an iterative process (SM); (6) finalizing of the coding framework, themes and sub-themes (all authors).

As per King’s (2012) recommendations, several quality checks were performed throughout the analysis process to enhance the quality of the data; (a) critical comparisons of codes, themes and sub-themes through weekly discussions among the team members and (b) the compilation of an audit trail to document the iterative changes to the coding framework over time.

## Results

### Sample characteristics

The final sample consisted of 45 parents aged between 28 to 51 years (M = 35.7; SD = 5.0; see Table 1 for complete demographic information of the sample). Notably, the sample was predominantly female (96%), married/ defacto (91%), tertiary educated (84%) and employed on either a full-time, part-time or casual basis (84%). Nevertheless, the age, gender and mobile-screen device usage of the target children of participants were relatively uniformly distributed.

**Table 1:** Sample demographic characteristics (*N* = 45)

Demographic Characteristic	N (%)
Parent gender	
Female	43 (95.6%)
Parent age (years)	
< 30	6 (13.3%)
31- 35	16 (35.6%)
36–40	16 (35.6%)
> 40	7 (15.6%)
Living arrangement	
Married/ defacto	41 (91.1%)
Highest level of education completed	
Secondary school (Year 11 or 12)	3 (6.7%)
TAFE/ Trade certificate/ diploma	4 (8.9%)
Undergraduate degree	17 (37.8%)
Post-graduate degree	21 (46.7%)
Employment	
Full-time	11 (24.4%)
Part-time/ Casual	24 (53.3%)
Maternity leave	3 (6.7%)
Not currently employed	7 (15.6%)
Household income (per annum)	
Up to $75,000	3 (6.7%)
$75,001 to 115,000	13 (28.9%)
$115,000 to 220,000	23 (51.1%)
$220,000 or more	6 (13.3%)
Residential location	
Urban area^#^	41 (91.1%)
Regional area^#^	4 (8.9%)
Number of children	
One child	15 (33.3%)
Two children	23 (51.1%)
Three or more children	7 (15.6%)
Target child age	
1 to < 3 years (toddler)	14 (31.1%)
3 to < 6 years (pre-schooler)	31 (68.9%)
Target child gender	
Female	18 (40%)
Target child's smartphone and tablet use	
Regular user	19 (42.2%)
Non-user	17 (37.8%)

Note. *N* = number of participants

^#^Of the ≈ 26 million population of Australia, ≈ 7.5 million (29%) live in regional areas while ≈ 17 million (69%) live in cities/ urban areas, with the rest of the population (2%) in rural and remote areas [47]

### Themes

**Fig. 2:**
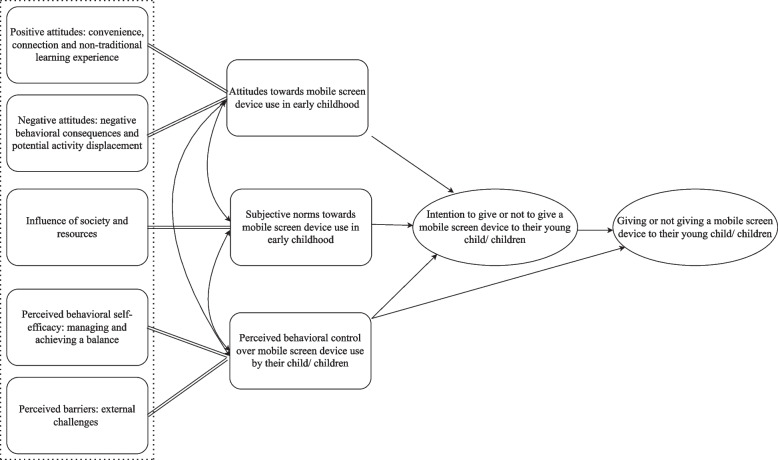
Mapping of the Themes to the A-priori Model of the Theory of Planned Behaviour

Five themes were coded using template analysis. The themes provided a concise summary of the attitudes, subjective norms and perceived behavioural control underpinning the motives for giving or not giving a device to a toddler or pre-schooler. Key themes and sub-themes are summarised in the following text, together with illustrative quotes describing each sub-theme. The visual mapping of the themes as they relate to the a-priori TPB model is illustrated in Fig. 2. A tabular summary of the sub-themes under each theme is further included as Additional File 3.

### Theme 1: Positive attitudes: Convenience, connection and non-traditional learning experience

For many parents, enabling their child to form and maintain connections with geographically distributed family and friends was a very common positive perception, which often influenced the decision to provide a mobile screen device (1.1 Keeping families connected). Parents also stated that this positive attitude towards screen use was intensified amid the ongoing COVID-19 pandemic, where restrictions limited opportunities for social interactions. Mobile screens often ensured the continuation of important family relationships:

“We’ve always had grandparents that lived a decent distance away, so she wouldn’t have a relationship with them if she couldn’t FaceTime” (Mother of a 2-yo, 42 yo, one child).

Parents also believed in the educational value of using a mobile screen device to foster their children’s learning and skills (1.2 Screens as the new teachers). Many parents used the terms “educational”, “interactive” or “learning” when describing positive use of mobile screens. They viewed screen use as beneficial insofar as it is a non-traditional learning experience which has the dual benefit of keeping their child engaged:

“I think we’re better off for having them…..he isn’t that interested in writing or drawing. Using an app gives him that opportunity to be interactive with his learning. And that’s… a real positive.” (Mother of a 2-yo, 43 yo, 4 children)

Some parents also believed that using these devices early in life equips young children to better face the foreseeable tech-focused future, especially in educational and employment settings (1.3 Digital future-proofing).

“I see more and more reliance on technology, and I feel it should probably be at a disadvantage more than an advantage by not having access to it and having the ability to use it successfully.” (Mother of a 3-yo, 36 yo, one child)

Conversely, several parents believed that using a device at an early age was not particularly advantageous as undoubtedly children would pick up the necessary skills as they grow older, given the intuitive nature of digital interfaces.

“But …like learning to use iPads in kindy [kindergarten] …if a one year old can learn to use it in a couple of days I don't think a 7, 8, 9, 10 year old is going to have any trouble with it, if my nearly 90 year old grandma can figure out how to use an iPad I don't know why you need to learn how to do it in kindy” (Mother of a 2-yo, 29 yo, 2 children)

In addition to the perceived benefits of device use, the convenience of providing a device to keep children occupied, especially for some “peace and quiet” or to complete daily chores during busy days, was a very common positive outcome evaluation (1.4 “A convenient babysitter”). Many parents who regularly used the device for this purpose mentioned that screens were very effective at keeping their child distracted or entertained and that without it parental exhaustion would be overwhelming.

“… because we're playing together and interacting all the other times of the day, that hour or two for me is the only chance to get other things done or just a break from “Mum, mum, mum, mum” and all these endless stories about everything.” (Mother of a 3-yo, 36 yo, one child)

While some parents viewed this option as “lazy” (Mother of a 1-yo, 34 yo, one child), some justified it as “pretty standard” (Mother of a 3-yo, 36 yo, one child). Several parents positively viewed the device as an effective way for their child to unwind and relax following a tiring or very active day (1.5 Digital downtime), for instance,

“if they're not napping, they still do need a bit of downtime, they're very busy little people.” (Mother of a 2-yo, 43 yo, 4 children).

### Theme 2: Negative attitudes: Negative behavioural consequences and potential activity displacement

Parents were concerned about devices supplanting real social interactions and opportunities for family bonding (2.1 There is no ‘real connection’):

"The worst bit is when .. you hand them your phone and then you’re like well now I’ve got nothing to do either. I’m just going to sit and stare at you playing on the phone." (Mother of a 4-yo, 37 yo, 2 children)

Some parents noted that screen experiences were not analogous to real-world experiences and that there was a risk that screen-version-of-life could dominate children’s worlds. This further ties in with the common notion many parents had around the opportunity costs of providing a device to a young child:

“Look out the window or we’ll have other activities, colouring in or books …and that’s what we use for stimulation, and we play games in the car, you know, like eye spy .. We overtly avoid screens.” (Father of a 5-yo, 40 yo, 2 children)

Parents were often regretful about the lost opportunities for traditional activities such as creative play, physical activity and hands-on exploration of natural environments due to the use of mobile screen devices (2.2 Old ways are the best ways). Some parents also believed in the value of boredom for their child and felt that screen time had become a ‘cure’ for boredom. Parents were concerned that a lack of boredom might mean that their child might not experience opportunities that foster their own creativity, innovative thinking and self-motivation for learning (2.3 “Boredom is a gift”):

“.. it’s just a really easy form of entertainment for them and if they get that too early on, I think then they haven’t developed that way of: (a) being bored, which is a talent to be okay with being bored; and then (b) to have internal intrinsic motivation to go find something to do or to make your own activity up or whatever.” (Mother of a 4-yo, 33 yo, one child)

Concerns among parents about significant patterns of unfavourable behavioural consequences they have observed, i.e., tantrums, aggressive or hyperactive behaviour, crying, following prolonged use of screens were highly prevalent (2.4 Agitated and overstimulated). It was a recurrent negative attitude towards providing mobile screen devices:

“.. when they have a lot of tablet time and TV time, they’re just rat bags (sic). ..They don’t listen, they’re really cranky, .. we really need to like.. ‘Okay, we’re going for a walk now.’ It sort of reset their brain a bit.” (Father of a 4-yo, 29 yo, 2 children)

Furthermore, some parents described using a device as a circumstantial distractor, but then that they lost track of time and facilitated more screen time than was intended. Parents reported that stopping use in these circumstances often led to tantrums which they found difficult to deal with and that they would rather not give the device at all to avoid dealing with post-device tantrums (Mother of a 2-yo, 31 yo, 2 children).

The persuasive nature of the mobile screen content often worried parents as they felt that the more their children used mobile screens, the more reliant on them they became (2.5 Hooked on screens). Numerous parents claimed screen content to be “addictive” or “overstimulating”, resulting in their children “getting engrossed” (Mother of a 4-yo, 42 yo, 4 children), “consumed” (Mother of a 4-yo, 34 yo, 2 children) or “completely absorbed” (Mother of a 1 -yo, 34 yo, one child):

“ Lots of colours, flashing lights, … they’re doing whatever they can to get an audience from people, and its immediate reactions, they don’t have to work hard for …a lot of content that they would be accessing.” (Mother of a 3-yo, 28 yo, one child)

They believed this attachment to the device and its content also stems from a growing sense of autonomy and agency by the child:

“..she’s at that age where she really wants to have the screen all the time and to hang onto it and to play with it and walk around with it.” (Mother of a 4-yo, 40 yo, 2 children).

The commercialisation of screen content including advertising and in-app purchases contributed to further worry about the age-appropriateness of content as “there’s so many adverts coming up that I don’t want him to have access to” (Mother of a 1-yo, 44 yo, one child). In particular, parents of younger children or toddlers were apprehensive about whether screen use at such an early age could be positive and meaningful given that their child lacked understanding about the nature of the device, as “they're just clicking away. That freaks me out.” (Mother of a 3-yo, 35 yo, one child). Parents felt as though their child was just attracted to the things that are “bright, colourful, cute and moving, the music and the cutesy voices” (Mother of a 3-yo, 35 yo, one child).

### Theme 3: Subjective norms: Influences of society and resources

Many parents reported their experiences of social pressures that influenced their decision-making with respect to early childhood device use. A salient reflection of many parents was the judgment and pressure they experienced from others (parents and non-parents) to manage their child’s behaviour, which resulted in seeking the most convenient pacifying option. In most cases, it was to give their child a mobile screen device (3.1 Parenting for other people’s comfort):

“… I felt really embarrassed, so I sat in the restaurant and apologised the whole way through the meal, .. I couldn’t – so I gave him my phone.” (Mother of a 1-yo, 34 yo, one child)

However, conversely, parents also commented that they had experienced judgment from others, particularly extended family members or friends, when their child was using a mobile screen in a public space:

“I don't really let him use it in public, more so because I’m worried about people judging me …which sounds really awful. … - say from my parents, they were .. like, ‘Oh, what are you doing?” (Mother of a 3-yo, 32 yo, 2 children)

Parents reported that this was often perceived as a “lazy” parenting option by others (Mother of a 1-yo, 34 yo, one child), which led them to believe so themselves. Parents often described this dilemma as “a double-edged sword” (Mother of a 2-yo, 28 yo, one child), “you’re damned if you do, you’re damned if you don’t” (Mother of a 4-yo, 33 yo, one child) or “you’re screwed either way” (Mother of a 2-yo, 32 yo, 2 children). The perception of social acceptability of the provision of mobile screens to young children was also a normative belief which drove the decision-making for some parents (3.2 Following the herd). While some termed it as “well, everybody else does it” or “succumbing to the majority” (Mother of a 4-yo, 40 yo, one child), there were a minority of parents who felt that “it would be great if every parent was on that same page; then there would be no pressure from other kids too…, there'd be no peer pressure” (Mother of a 5-yo, 41 yo, 4 children).

In addition to influences from family and community, the influence of resources and conflicting information was also frequently talked about (3.3 Mixed messages and urban myths):

“And on the one hand, everything’s saying screen time’s not great for kids, that it’s dangerous, there’s all this content. But on the other hand, schools are saying, ‘You must do it this way’. It doesn’t seem to fit together.” (Mother of a 4-yo, 40 yo, 2 children)

Parents reported that the different viewpoints and arguments around benefits and risks of mobile screens, the uncertainties, the rigidity around health messaging on screen time (disregarding certain nuances) seemed confusing. It led some parents to take an unbalanced (i.e., either too strict or too relaxed) outlook on providing mobile screens to young children. They mention that this has resulted a wide spectrum of views within the parent community:

“So, I think many parents are looking towards those guidelines on how much screen time you should be doing … some other people probably totally ignore that and just do whatever they want to do. .. it’s quite variable really from what I’ve seen with other parents.” (Mother of a 4-yo, 36 yo, 3 children)

### Theme 4: Perceived behavioural self-efficacy: Managing and achieving a balance

Parents’ perceived self-efficacy or confidence in their own decision-making to provide and appropriately manage the use of mobile screens further influenced their motives to provide or not provide a device. Most parents detailed various ways in which they ensured that they have some level of control over giving a device to their child and the use that followed (4.1 Internal and external locus of control). There were two main ways parents attributed control; (a) internal—actively or directly assuming control themselves (e.g., setting limits or parental controls); or (b) external—attributing control to indirect or external factors (e.g., letting the device run out of charge, hiding devices). When discussing their perceived self-efficacy, parents commonly used phrases such as “strict boundaries” (Mother of a 3-yo, 36 yo, one child), “resisting the requests” (Mother of a 4-yo, 33 yo, 2 children) or “rules and limits” to discuss an internal locus of control:

“… me and my husband we've got pass codes on our phone so they can't access them without that. At the moment the wi-fi is also disconnected ..” (Mother of a 4-yo, 45 yo, 3 children)

Meanwhile phrases including “out of sight, out of mind” (Mother of a 4-yo, 40 yo, 2 children), “oh it’s broken” (Mother of a 4-yo, 37 yo, 2 children) or “the battery is dead/ flat” described screen time management strategies which relied on an external locus of control:

“.. I let it almost run out and I don’t charge it. .. if her battery runs out because she’s only had 20 per cent and that’s all I’ve bothered to charge it, .., “oh, sorry, Darling, there’s no more iPad.” (Mother of a 3-yo, 36 yo, one child)

Many parents conceded that their own screen use and modelling plays a big role in their child’s usage habits and device-related requests, and that it is important to be mindful of role-modelling. Many parents stated that their awareness of their own problematic screen time habits sometimes led them to be stricter with their child’s device use (4.2 ‘I feel like a hypocrite’):

“I know how addictive screens can be and that’s one of the reasons I don’t want my kids started.” (Mother of a 4-yo, 39 yo, 2 children)

They used phrases such as “double-standard” (Mother of a 1-yo, 44 yo, one child) and “do as I say, not as I do” (Mother of a 3-yo, 31 yo, 2 children) to highlight the hypocrisy of resisting screen time requests from their child despite feeling that their own screen use is somewhat questionable.

### Theme 5: Perceived barriers: External challenges

Parents discussed several perceived external barriers to appropriately providing a device and effectively managing healthy mobile screen habits. The most noteworthy barrier was simply the reality of life (5.1 Ideal meets reality). For most parents, life was busy and messy, and similar to things such as their child’s eating and sleeping routines, screen time was another part of parenting that did not go the way they thought it would. Parents reported that screen time was “getting in the way” of them being they type of parent they had thought that they would be or was making them “fall short of the ideal” (Father of a 4-yo, 29 yo, 2 children). There was often acceptance of this reality, where what they imagined to be ideal or perfect parenting in relation to their child’s mobile screen use was in fact, quite different in truth. What comprised the ideal varied among parents, however many mentioned that while zero screen time would be ideal, it was also impossible. Some parents disagreed with the notion of ideal and thought it would largely depend on the family context and circumstances. Interestingly, some parents noted how their perceptions of ideal drastically changed following the birth of their child:

“..before you have children where you go, “Oh no, I’m not going to let my children play on screens and have access to my phone. Then you get there…, “Holy crap, giving him my phone for five minutes while I am at the doctor’s appointment so he’s quiet and not screaming in my face is actually really handy.” .. going through it has definitely changed my idea of what is okay and what is realistic” (Mother of a 2-yo, 32 yo, 2 children)

Many parents expressed their unease around the predicament of considering the costs versus benefits to their own wellbeing of providing a device (5.2 A terrible trade-off). While the device was a helpful and convenient way to alleviate the burden or exhaustion of parenting, the trade-off was that they felt guilty that their child often used the device for much longer than they would prefer. However, some claimed that if the perceived behavioural and developmental costs of prolonged use meant that they did not provide their child with a device, it often affected their ability to “just get through the day” (Mother of a 2-yo, 38 yo, one child). Another factor which contributed to this dilemma was the pervasive nature of mobile screens across families, and how wide-spread and “mainstream” it is (5.3 Holding back the tide). Some parents commented on how effortful it is to be the minority and to not “succumb to the majority” (Mother of a 4-yo, 40 yo, one child):

“.. it’s really hard for parents to be able to make an informed choice…a lot of the choice is taken away by the fact that it’s so mainstream now.” (Mother of a 4-yo, 33 yo, 2 children)

Incompatibilities in views within family members and varying family circumstances (e.g., multiple children, urban -versus regional living) were also often apparent barriers to assertive decision-making and management of young children’s mobile screen use (5.4 Family matters):

“.. if we had another child or something like that, and I was trying to look after a baby and feed a baby and change nappies …, I suspect I'd probably be using it a little bit more. And it really annoys me when my husband is checking his phone, and I feel like saying to him, "You're not hearing a word I'm saying", but then he would say the same thing about me.”(Mother of a 2-yo, 38 yo, one child)

Nevertheless, some parents also felt very supported within their families through regular conversations and “being on the same page” (Mother of a 1-yo, 29 yo, one child):

“I just get that mum rage big time, so it does affect me. But my husband’s very supportive. We have a plan. We assume it will get better before we do it... just try and debrief through.” (Mother of a 4-yo, 33 yo, one child)

## Discussion

Our study aimed to qualitatively explore the factors that underpin parents’ motives for provision of smartphones and tablets to toddlers and pre-schoolers through the lens of the Theory of Planned Behaviour. There are several interesting findings that may help to inform current screen time parenting practices and guide future research. First, we found that a spectrum of attitudes, subjective norms and perceived behavioural control underpin the varied motives of parents for providing or not providing mobile screen devices to young children in their everyday lives. The inter-related nature of the themes gives a clear picture of the factors that drive parent decision-making and is consistent with the general structure and mechanisms described by the TPB [33]. Second, in addition to the conventional TPB processes, a bi-directional relationship between attitudes and perceived behavioural control seemed to be instrumental in determining parents’ motives and behaviour towards mobile screens in early childhood, one which appeared to be substantially influenced by subjective norms. Parents described a sense of incongruence that they associated with over-bearing subjective norms suppressing their own attitudes and perceived behavioural control with respect to managing screen use. The collective effect of these interactions resembled cognitive dissonance, whereby parents were realigning their attitudes with their actual behaviours rather than aligning their behaviours with existing attitudes. Third, the qualitative insights suggest that many parents’ views and experiences are dependent on household composition variables (e.g., multiple children) and their socio-economic circumstances (e.g., urban or regional location, employment, familial support). Finally, the context-dependency of parental decision-making, along with individual differences in beliefs and attitudes is likely to reduce the effectiveness of universal and rigid health messaging around early childhood screen time guidelines.

While there has been relatively limited qualitative work in parental decision making relating to screen time, the findings of the present study offer rich and in-depth insights into the quantitative research that examine factors underpinning parent decision making in relation to early childhood screen use. For instance, the present findings align well with past studies (e.g., 24, 36) in which a large proportion of variance (38 – 50%) in the screen time decision-making of parents of young children was explained by the TPB psychosocial factors. Further, the power and heavy influence of parental attitudes in child use of mobile screens [48] and total screen time [13, 20], have been consistently demonstrated in previous early childhood screen use research. As this field of work is often characterised by polarised debate and narrative [49], qualitative research is especially important to provide context and a human voice to the factors which underpin parental views and perceptions.

Parents’ attitudes about mobile screens ranged from positive views about the convenience, pragmatism and usefulness as a teaching tool, to negative views relating to potential overstimulation, displacement of developmentally healthy activities, social interactions and hinderance of creativity among children. These attitudes are consistent with qualitative research on use in early childhood [30], as well as middle-childhood [50], where parents reported feeling tension between the benefits and harms of mobile devices to children’s development. In our study, parents appeared to experience varying degrees of incongruence when their attitudes towards how they planned to manage screen time conflicted with their perceived self-efficacy to do so. Parents’ subjective norms (comprising the influence of family, society, and information) appeared to suppress, or possibly negate, the influence of their own attitudes on their level of device-related perceived behavioural control (including their self-efficacy). For instance, parental self-efficacy with respect to device provision was largely affected by the burdens of parenting and the reality of life. The perceived barriers made it very challenging for parents to do what they believe is ideal and to hold back the tide in terms of managing when and where they would permit access to a device. These decisions elicited tensions and dilemmas for parents as they weighed the costs and benefits to their child and to their own wellbeing. The challenges of experiencing such cognitive conflict have been consistently illustrated in previous qualitative research on parental perceptions around use in early [30, 51, 52] and middle childhood, as well as adolescence [53]. As a result, differing levels of cognitive dissonance were evident among parents, which subsequently led them to realign their attitudes and beliefs with their perceived behavioural control and behaviours, in order to reduce the discomfort and tension they felt. For instance, they would rationalize their behaviour as ‘being pretty standard’ or as ‘what the majority does’.

In addition to the ‘at home’ pressure and dilemmas parents face in everyday device-related decision-making, perceived subjective norms also seemed to evoke a great deal of negative emotions and self-evaluations among parents. For instance, high internalised guilt, fear, worry and regrets stemming from the pressure and judgment from family and society underpinned screen-related parenting practices that were aimed at managing young children to make others feel comfortable. These perceived pressures, parental guilt and anxiety are particularly in congruence with qualitative evidence in recent studies where parents report feeling overwhelmed as they navigate the societal, social and individual expectations around managing screen time [49, 52]. In the present study, parents commonly reported the paradoxical nature of the social expectations that drove their decision-making in public places. On one hand, parents felt pressure to keep their child relatively quiet and ‘under control’ in social settings, but on the other hand felt judged by others for achieving the goal of a quiet child via the use of a screen. The conflicting social expectations and fear of negative evaluations by others (known and unknown) may have a direct impact on perceived behavioural control for parents. Not knowing which course of action is the ‘right’ one could significantly reduce parenting self-efficacy and sense of control.

It is especially important to view the results of the present study in light of the COVID-19 pandemic. The pandemic very likely influenced perspectives on the role of technology in everyday lives of young children. For instance, COVID-19 lockdowns and online learning from home have warranted being technologically active to engage and complete any form of learning for children. Over time, these changes have contributed to the normalizing of use of screen-based devices as educational tools for young children, with growing acceptance of their role in learning [54]. This is evident through the increased rates of screen use among families with young children across the world [6], including Australia, [55] since the commencement of the pandemic.

Our findings have implications for the development of early childhood screen time guidelines. The themes that reflect parental difficulties and cognitive dilemmas with respect to managing screen time decision-making can be used as a starting point for developing more family-friendly and realistic screen time guidelines. Parental and social attitudes, social norms and parental self-efficacy with respect to behavioural control are all potential areas for healthy screen time intervention. Rather than largely unrealistic and dispiriting screen time guidelines, parents and caregivers would benefit from practical, consistent and positive support mechanisms. We propose several changes that may help to achieve that aim. First, parents require more flexible and feasible solutions to help reduce their perceived lack of alternatives to mobile screens in various stressful or busy scenarios. As a starting point, we suggest encouraging more no-tech alternatives (e.g., arts and crafts; photo albums) or low-tech alternatives (e.g., audiobooks or podcasts), along with ways to support parents to know how to promote more intentional technology use at home (e.g., mindfulness about the content and duration). Second, it is important to move beyond the dichotomous ‘good or bad’ argument of technology use in early childhood when engaging with parents regarding screen time. Instead, parents need to be empowered to raise digitally healthy children, and to do so will require effective resources that help them decide what is ‘good use’ and ‘good content’. Platforms such as Common Sense Media already offer resources aiding decision-making with age-appropriate content, however parents only benefit from these resources if they go looking for, and happen to find, appropriate online resources. A more visible public health program for parents may help to increase awareness of such support platforms. Third, health messaging should attempt to debunk myths which often result in parent misperceptions, such as the idea that children would fall behind in school if technology is not introduced as early as possible. Many parent attitudes and behaviours with respect to mobile screens appear to be driven by emotions, typically fear and guilt. By actively dispelling various kinds of screen time myths, parents may have a better chance of making positive, healthy decisions rather than fear-based ones.

While the present study gave voice to parents and provided detailed parenting-screen time insights, some limitations must be acknowledged. First, the nature of the topic (parenting and screen time) may have led parents that were confident in their decisions to volunteer for participation in the larger longitudinal study, while parents who felt their screen time-parenting was, in their own estimation, poor, may have avoided participating for fear of judgment. While the research team were extremely careful to avoid any language that could potentially be perceived as judgmental, we accept that parenting is a difficult subject and that some parents may have elected to not participate for this reason. The method of data collection (i.e., online via Zoom) may have also resulted in parents who are more confident with their use of online media to opt in for the interviews. This implies a certain closeness of the participants to digital media and devices, which in turn may be influencing their attitudes towards their children using mobile-screen devices. Second, parent gender, ethnicity, residential location and socio-economic status (SES) were not uniformly distributed across the sample despite the use of a sampling matrix. The non-uniformity may have affected the transferability of the results to diverse families across Australia (e.g., first nation families, parents of a child with a disability, remote or rural families). Given that demographic characteristics such as the SES have been shown to be associated with young children’s screen time (e.g., [56, 57]), demographic factors are likely to also influence parents’ attitudes and perceived behavioural control, which warrants further investigation.

## Conclusions

The current study investigated parental decision-making related to use of mobile screens in early childhood using the Theory of Planned Behaviour framework. Attitudes, subjective norms and perceived behavioural control collectively explained parent motives for providing mobile screens to young children. Further, varying levels of attitude-behavioural incongruence and cognitive dissonance were experienced by parents when decision-making, possibly due to the dominating influence of subjective norms. The increased acceptance of the pervasiveness of mobile technology in the post-COVID era is a prominent change among families. Future research could further explore how family practices have changed as a result of increased pandemic-related screen use and whether or not there has been any impact on child developmental outcomes over time. Parents’ experience of cognitive dissonance and associated emotions also present avenues for future research, particularly with respect to targets for interventions aimed at supporting and empowering parents to raise their children to be healthy digital citizens. Most importantly, we acknowledge the need for more tailored resources offering no- and low-technological alternatives and practical health messaging for parents addressing provision of mobile screen devices in early childhood. Finally, we encourage public health policy makers to engage in co-design approaches with parents in order to formulate more realistic and user-friendly parenting resources.

## Supplementary Information

**Additional file 1.** 

**Additional file 2.** 

**Additional file 3.**

## Data Availability

Due to the nature of qualitative interviews, the data are not publicly available however can be made available upon request from the corresponding author [SM].

## Abbreviations

TPB: Theory of Planned Behaviour
SES: Socio-economic status

## References

[1] Rideout V (2017). The Common Sense census: Media use by kids age zero to eight.

[2] Rideout V (2020). The Common Sense Census: Media Use by Kids Age Zero to Eight.

[3] Radesky JS, Weeks HM, Ball R, Schaller A, Yeo S, Durnez J (2020). Young children’s use of smartphones and tablets. Pediatrics.

[4] Eales L, Gillespie S, Alstat RA, Ferguson GM, Carlson SM (2021). Children’s screen and problematic media use in the united states before and during the covid-19 pandemic. Child Dev.

[5] Olive L, Sciberras E, Berkowitz TS, Hoare E, Telford R, Mikocka-Walus A, et al. Child and parent physical activity, sleep and screen time during COVID-19 compared to pre-pandemic nationally representative data and associations with mental health. 2020. 10.3389/fpsyt.2021.774858 PMC8886612 35242059

[6] Bergmann C, Dimitrova N, Alaslani K, Almohammadi A, Alroqi H, Aussems S (2022). Young children’s screen time during the first COVID-19 lockdown in 12 countries. Sci Rep.

[7] Radesky JS, Schumacher J, Zuckerman B (2015). Mobile and interactive media use by young children: the good, the bad, and the unknown. Pediatrics.

[8] Mallawaarachchi SR, Anglim J, Hooley M, Horwood S (2022). Associations of smartphone and tablet use in early childhood with psychosocial, cognitive and sleep factors: a systematic review and meta-analysis. Early Childhood Research Quarterly.

[9] Domoff SE, Borgen AL, Radesky JS. Interactional theory of childhood problematic media use. Human Behavior and Emerging Technologies. 2020. 10.1002/hbe2.217 PMC9645666 36381426

[10] Kabali HK, Irigoyen MM, Nunez-Davis R, Budacki JG, Mohanty SH, Leister KP (2015). Exposure and use of mobile media devices by young children. Pediatrics.

[11] Jago R, Sebire SJ, Lucas PJ, Turner KM, Bentley GF, Goodred JK (2013). Parental modelling, media equipment and screen-viewing among young children: cross-sectional study. BMJ Open.

[12] McDaniel BT (2019). Parent distraction with phones, reasons for use, and impacts on parenting and child outcomes: a review of the emerging research. Human Behavior and Emerging Technologies.

[13] Lauricella AR, Wartella E, Rideout VJ (2015). Young children's screen time: the complex role of parent and child factors. J Appl Dev Psychol.

[14] Xu H, Wen LM, Rissel C (2015). Associations of parental influences with physical activity and screen time among young children: a systematic review. J Obes.

[15] McCloskey M, Johnson SL, Benz C, Thompson DA, Chamberlin B, Clark L (2018). Parent perceptions of mobile device use among preschool-aged children in rural head start centers. J Nutr Educ Behav.

[16] Konok V, Bunford N, Miklósi Á (2020). Associations between child mobile use and digital parenting style in Hungarian families. J Child Media.

[17] Eyimaya AO, Irmak AY (2021). Relationship between parenting practices and children's screen time during the COVID-19 Pandemic in Turkey. J Pediatr Nurs.

[18] Tang L, Darlington G, Ma DW, Haines J (2018). Mothers’ and fathers’ media parenting practices associated with young children’s screen-time: a cross-sectional study. BMC obesity.

[19] Hinkley T, McCann JR (2018). Mothers’ and father’s perceptions of the risks and benefits of screen time and physical activity during early childhood: a qualitative study. BMC Public Health.

[20] Miguel-Berges ML, Santaliestra-Pasias AM, Mouratidou T, Flores-Barrantes P, Androutsos O, De Craemer M (2020). Parental perceptions, attitudes and knowledge on European preschool children’s total screen time: the ToyBox-study. Eur J Pub Health.

[21] Vittrup B, Snider S, Rose KK, Rippy J (2016). Parental perceptions of the role of media and technology in their young children’s lives. Journal of Early Childhood Research.

[22] Veldhuis L, van Grieken A, Renders CM, HiraSing RA, Raat H (2014). Parenting style, the home environment, and screen time of 5-year-old children; the ‘be active, eat right’study. PLoS ONE.

[23] Detnakarintra K, Trairatvorakul P, Pruksananonda C, Chonchaiya W (2020). Positive mother-child interactions and parenting styles were associated with lower screen time in early childhood. Acta Paediatr.

[24] Carson V, Janssen I (2012). Associations between factors within the home setting and screen time among children aged 0–5 years: a cross-sectional study. BMC Public Health.

[25] Jago R, Wood L, Zahra J, Thompson JL, Sebire SJ (2015). Parental control, nurturance, self-efficacy, and screen viewing among 5-to 6-year-old children: a cross-sectional mediation analysis to inform potential behavior change strategies. Child Obes.

[26] Nevski E, Siibak A (2016). The role of parents and parental mediation on 0–3-year olds’ digital play with smart devices: Estonian parents’ attitudes and practices. Early years.

[27] Papadakis S, Zaranis N, Kalogiannakis M (2019). Parental involvement and attitudes towards young Greek children’s mobile usage. International Journal of Child-Computer Interaction.

[28] McCloskey M, Thompson DA, Chamberlin B, Clark L, Johnson SL, Bellows LL (2018). Mobile device use among rural, low-income families and the feasibility of an app to encourage preschoolers’ physical activity: qualitative study. JMIR pediatrics and parenting.

[29] O’Connor J, Fotakopoulou O (2016). A threat to childhood innocence or the future of learning? Parents’ perspectives on the use of touch-screen technology by 0–3 year-olds in the UK. Contemp Issues Early Child.

[30] Radesky JS, Eisenberg S, Kistin CJ, Gross J, Block G, Zuckerman B (2016). Overstimulated consumers or next-generation learners? Parent tensions about child mobile technology use. The Annals of Family Medicine.

[31] Magilvy JK, Thomas E (2009). A first qualitative project: qualitative descriptive design for novice researchers. J Spec Pediatr Nurs.

[32] Co JP, Perrin JM (2005). Qualitative research and ambulatory pediatrics. Ambul Pediatr.

[33] Ajzen I (1991). The theory of planned behavior. Organ Behav Hum Decis Process.

[34] Hamilton K, van Dongen A, Hagger MS (2020). An extended theory of planned behavior for parent-for-child health behaviors: a meta-analysis. Health Psychol.

[35] Hamilton K, Thomson CE, White KM (2013). Promoting active lifestyles in young children: Investigating mothers’ decisions about their child’s physical activity and screen time behaviours. Matern Child Health J.

[36] Hamilton K, Spinks T, White KM, Kavanagh DJ, Walsh AM (2016). A psychosocial analysis of parents' decisions for limiting their young child's screen time: an examination of attitudes, social norms and roles, and control perceptions. Br J Health Psychol.

[37] World Health Organization (2018). Nurturing care for early childhood development: a framework for helping children survive and thrive to transform health and human potential.

[38] Radesky JS, Christakis DA (2016). Increased screen time: implications for early childhood development and behavior. Pediatr Clin.

[39] King N, Brooks J. 14 THEMATIC ANALYSIS IN ORGANISATIONAL RESEARCH. The Sage handbook of qualitative business and management research methods. 2021:201.

[40] King N, Brooks JM. Template analysis for business and management students: Sage; 2017.

[41] Berger R (2015). Now I see it, now I don’t: Researcher’s position and reflexivity in qualitative research. Qual Res.

[42] Evans S, Mikocka-Walus A, Klas A, Olive L, Sciberras E, Karantzas G, et al. From “It has stopped our lives” to “Spending more time together has strengthened bonds”: The varied experiences of Australian families during COVID-19. Frontiers in psychology. 2020:2906. 10.3389/fpsyg.2020.588667 PMC7606874 33192922

[43] Brooks J, King N. Qualitative psychology in the real world: the utility of template analysis. 2012. 10.1080/14780887.2014.955224 PMC4960514 27499705

[44] Braun V, Clarke V (2021). Can I use TA? Should I use TA? Should I not use TA? Comparing reflexive thematic analysis and other pattern-based qualitative analytic approaches. Couns Psychother Res.

[45] Brooks J, McCluskey S, Turley E, King N (2015). The utility of template analysis in qualitative psychology research. Qual Res Psychol.

[46] King N (2012). Doing template analysis. Qualitative organizational research: Core methods and current challenges.

[47] Australian Bureau of Statistics. Population: Census. 2021.

[48] Pila S, Lauricella AR, Piper AM, Wartella E (2021). The power of parent attitudes: examination of parent attitudes toward traditional and emerging technology. Human Behavior and Emerging Technologies.

[49] Livingstone S, Franklin K (2018). Families with young children and ‘screen time’. Journal of Health Visiting.

[50] Solomon-Moore E, Matthews J, Reid T, Toumpakari Z, Sebire SJ, Thompson JL (2018). Examining the challenges posed to parents by the contemporary screen environments of children: a qualitative investigation. BMC Pediatr.

[51] Radesky JS, Kistin C, Eisenberg S, Gross J, Block G, Zuckerman B (2016). Parent perspectives on their mobile technology use: the excitement and exhaustion of parenting while connected. J Dev Behav Pediatr.

[52] Findley E, LaBrenz CA, Childress  S, Vásquez‐Schut G , Bowman  K (2022). ‘I’m not perfect’: navigating screen time among parents of young children during COVID‐19. Child Care Health Dev.

[53] Barroso CS, Springer AE, Ledingham CM, Kelder SH (2020). A qualitative analysis of the social and cultural contexts that shape screen time use in Latino families living on the US-Mexico border. Int J Qual Stud Health Well Being.

[54] Hu X, Chiu MM, Leung WMV, Yelland N (2021). Technology integration for young children during COVID-19: Towards future online teaching. Br J Edu Technol.

[55] Ribner AD, Coulanges L, Friedman S, Libertus ME, Hughes C, Foley S (2021). Screen time in the Coronavirus 2019 Era: international trends of increasing use among 3-to 7-year-old children. J Pediatr.

[56] Carson V, Spence JC, Cutumisu N, Cargill L (2010). Association between neighborhood socioeconomic status and screen time among pre-school children: a cross-sectional study. BMC Public Health.

[57] Tandon PS, Zhou C, Sallis JF, Cain KL, Frank LD, Saelens BE (2012). Home environment relationships with children’s physical activity, sedentary time, and screen time by socioeconomic status. Int J Behav Nutr Phys Act.

