# Supplementary material for: “You’re damned if you do, you’re damned if you don’t”: a qualitative exploration of parent motives for provision of mobile screen devices in early childhood
Source: BMC Public Health. 2022 Nov 2;22:2011. doi: 10.1186/s12889-022-14459-0 (PMC9629764; doi:10.1186/s12889-022-14459-0)
Supplement: Supplementary file 2 — Additional file 2. [file 12889_2022_14459_MOESM2_ESM.docx]

## Additional File 2

## Interview Schedule A

1.

1. What would ideal use of a smartphone/tablet by a toddler or preschooler look like to you?

- Including length of use per day or per week?
- Including content?

1. How do your ideas about ideal use compare to the actual use in your home?
2. How do you think parents decide what is appropriate content or activities on screens for their child? (e.g., websites they could visit or apps they could use)
3. What things do you think are good or bad about young children using smartphones or tablets?

- Can you tell me about any benefits that you see in providing a toddler or preschooler a smartphone/ tablet?
- What aspects of use do you think are most helpful for toddlers or preschoolers?
- Can you tell me about any concerns or reservations, if any, that you have about toddlers or preschoolers using them?

1. What do you think are things that make toddlers or preschoolers want to stay on their smartphone/tablet?

2.

1. Why do you think parents provide a smartphone/ tablet to their toddler or preschooler to use?

- Do you think any of those reasons affected you in your decisions?
- Is there an age that you think is ideal for children to have their own smartphone/tablet? What and why is that the ideal age?

1. [for users] Think back to when you first allowed <child’s name> to use a smartphone/ tablet, can you describe your own decision-making process at that time?
   - Was that an active decision or was it happenstance/ a coincidence?
   - How about the first time they were allowed to use in public?

[for non-users] When do you foresee as a time that <child’s name> might be allowed to use screens?

- Can you describe how you made that decision?

1. What would your child’s likely response be if they were suddenly unable to use their device for a while (e.g., if it broke)?

- What would you do in that situation?

1. What do you think life would be like if smartphones or tablets were not invented at all?
2. Is there anything else that you’d like to add?

## Interview Schedule B

1. (a) What would ideal use of a smartphone/tablet by a toddler or preschooler look like to you?

- Including length of use per day or per week?
- Including content?

1. How do your ideas about ideal use compare to the actual use in your home?

(c) How do you think parents decide what is appropriate content or activities on screens for their child? (e.g., websites they could visit or apps they could use)

- What strategies do you use yourself?
- How did you come up with those strategies?
- How often do you revisit your choice of appropriate content?

1. Can you tell me how you manage screen use in your home?
   - Can you describe any screen time rules that you have in your house?
   - How did you come up with the rules/ strategies?
   - What are the challenges that you face at home when managing screen time?
   - How does <child’s name> react to you imposing rules around screen time?
   - What do you think are challenges that other parents may be facing when managing screen time for their kids?
2. [If regular user] How, if at all, do you monitor <child’s name>’s use of a smartphone or tablet?

[If not a regular user] How would you monitor <child’s name>’s smartphone or tablet use in the future?

1. How do you feel about maintaining control of <child’s name>’s device use as they grow up? [if parent of multiple children] If it makes it easier, you could consider your experience with your older kids when you answer this

- What things do you think will change?
- What things, if any, do you worry about with respect to screen use as <child’s name> grows up?

1. Where would you go, or who would you talk to, if you wanted to find out information about managing screen time (or about healthy screen time guidelines)?

- How useful was the information that you found?
- How accessible was such information?
- Are there any health professionals or other people you would like to talk to about your child’s screen time/ screen time in general?
- [If no] Where do you think you’d go if you wanted to find such information?

1. What do you think would help you in managing your child’s/ <children’s name>’s screen time (present and/or future)?
   - In an ideal world, what would be the best way to help you manage their screen time?
2. Are there any screen time guidelines for toddlers or preschoolers that you are aware of?

- What do you understand them to be?
- [If not aware] Would you be interested in finding out about them?

[If yes to finding out] Just to give you a quick summary of the WHO guidelines, for kids aged 2 to 5 years, screen time (inclusive of all media) should be limited to less than one hour per day, and shared screen time is recommended. For kids younger than 2 years, there should be no screen time other than video chatting. No screens are recommended during meals and 1 hour before bedtime.

- What are your thoughts about them?
- How much are you able to adhere to them? How easily are you able to adhere to them?
- Is there anything else that you think would be useful as part of these guidelines?

That’s all the formal questions we have, is there anything else that you think is important that you’d like to talk about today?
