# Supplementary material for: “You’re damned if you do, you’re damned if you don’t”: a qualitative exploration of parent motives for provision of mobile screen devices in early childhood
Source: BMC Public Health. 2022 Nov 2;22:2011. doi: 10.1186/s12889-022-14459-0 (PMC9629764; doi:10.1186/s12889-022-14459-0)
Supplement: Supplementary file 3 — Additional file 3. [file 12889_2022_14459_MOESM3_ESM.docx]

**Additional File 3**

Themes and Sub-theme Descriptions with Illustrative Quotes from Participants

| Sub-theme | Description | Illustrative Quotes |
| --- | --- | --- |
| **Theme 1: Positive attitudes towards mobile screens: Convenience, connection and non-traditional learning experience** | | |
| 1.1 Keeping families connected | Devices enable forming connections and maintaining relationships | “..we only took him back when he was 6 months, but he’s able to .. have that relationship with his great-grandmother now. Which when I was growing up, we didn’t have that because we had to only speak on the phone. .... it’s opened our doors for communication with people all over the world” (Mother of a 4-yo, 40 yo, one child) |
| 1.2 Screens as the new teachers | Learning through mobile screens offer fun, non-traditional, and more engaging forms of learning things they otherwise could not through books or other means | “I think we're better off for having them….. he isn't that interested in writing or drawing. Using an app gives him that opportunity to be interactive with his learning. And that's… a real positive.” (Mother of a 4 yo, 43 yo, 4 children) |
| 1.3 Digital future-proofing | Using devices from a young age will aid children to be well-equipped with skills to navigate the digital-focused future | “I see more and more reliance on technology, and I feel it should probably be at a disadvantage more than an advantage by not having access to it and having the ability to use it successfully.” (Mother of a 3-yo, 36yo, one child) |
| 1.4 “A convenient babysitter” | Devices as a helpful tool for keeping children occupied in specific circumstances (e.g., vaccinations, air travel) and in day-to-day life | [Specific circumstances]”I have given him my phone once when he went to a podiatrist. … I thought he was going to flip out with them having to dig out his toenail …, so he did once get that and it worked a charm….” (Mother of a 4-yo, 33 yo, one child)  [Daily activities] “… because we're playing together and interacting all the other times of the day, that hour or two for me is the only chance to get other things done or just a break from “Mum, mum, mum, mum” and all these endless stories about everything.” (Mother of a 3-yo, 36 yo, one child) |
| 1.3 Digital downtime | Devices as a helpful tool for children to relax after an active day | “She fits so much into her day, I don’t feel that that’s a problem for her. … I’m looking out the window now, and she’s literally hanging from the monkey bars. So it’s not like it’s taking away her time from being physically active and all the rest of it.” (Mother of a 4-yo, 40 yo, 2 children) |
| **Theme 2: Negative attitudes towards mobile screens: Negative behavioral consequences and potential displacement through mobile screens** | | |
| 2.1 There is no ‘real’ connection | Concerns that providing devices limit opportunities for communication and social interactions | "The worst bit is when .. you hand them your phone and then you’re like well now I’ve got nothing to do either. I’m just going to sit and stare at you playing on the phone." (Mother of a 4-yo, 37 yo, 2 children) |
| 2.2 The old ways are the best ways | Preference for traditional developmentally healthy activities such as play, reading and physical activity | Look out the window or we’ll have other activities, colouring in or books …and that’s what we use for stimulation, and we play games in the car, you know, like eye spy .. We overtly avoid screens. (Father of a 5-yo, 40 yo, 2 children) |
| 2.3 “Boredom is a gift” | Concern that using mobile screens whenever children are bored is hindering their imagination | “Getting your child to be bored enough where they have to create their own fun, rather than just, ‘Oh, you’re bored. Okay, here’s something else to do.’ … and with a tablet, they’re never going to get that because for most apps, … the app creates the fun” (Father of a 4-yo, 29 yo, 2 children) |
| 2.4 Agitated and overstimulated | Negative experiences of unfavourable behavioral consequences following prolonged use of mobile screens | “.. when they have a lot of tablet time and TV time, they’re just rat bags. ..They don’t listen, they’re really cranky, .... ‘Okay, we’re going for a walk now.’ It sort of reset their brain a bit.” –(Father of a 4-yo, 29 yo, 2 children) |
| 2.5 Hooked on screens | Concerns about the addictive nature of the mobile screen content and its features which results in an attachment to the device. | “ Lots of colours, flashing lights, … they’re doing whatever they can to get an audience from people, and its immediate reactions, they don’t have to work hard for …a lot of content that they would be accessing.” (Mother of a 3-yo, 28 yo, one child) |
| **Theme 3: Subjective norms on providing or not providing mobile screen : Influences of society and resources** | | |
| 3.2 Parenting for other people’s comfort | Judgment from others as a strong driver of providing or not providing a device. | [Providing] “… I felt really embarrassed, so I sat in the restaurant and apologised the whole way through the meal, .. I couldn’t – so I gave him my phone.” (Mother of a 1-yo, 34 yo, one child)  [Not providing] “I don't really let him use it in public, more so because I’m worried about people judging me …which sounds really awful. … - say from my parents, they were .. like, ‘Oh, what are you doing?” (Mother of a 3-yo, 32 yo, 2 children) |
| 3.2 Following the herd | Giving mobile screens to young children have become a common practice among a majority of parents | “… it's just socially … acceptable more and more so that's – if such-and-such is doing it, it must be okay for me to do it too” (Mother of a 4-yo, 38 yo, only child) |
| 3.3 Mixed messages and urban myths | Conflicting information confuses parents’ decisions on whether or not to provide devices | “And on the one hand, everything’s saying screen time’s not great for kids, that it’s dangerous, there’s all this content. But on the other hand, schools are saying, ‘You must do it this way’. It doesn’t seem to fit together.” (Mother of a 4-yo, 40 yo, 2 children) |
| **Theme 4: Perceived behavioral self-efficacy and control related to providing mobile screens: Managing and achieving a balance** | | |
| 4.1 Internal and external locus of control | Parents attempt to manage their child’s use in two ways: (1) internal; and (2) external factors | [Internal] “… me and my husband we've got pass codes and our phone so they can't access them without that. At the moment the wi-fi is also disconnected ..” (Mother of a 4-yo, 45 yo, 3 children)  [External] “.. I let it almost run out and I don’t charge it. .. if her battery runs out because she’s only had 20 per cent and that’s all I’ve bothered to charge it, .., “oh, sorry, Darling, there’s no more iPad.” (Mother of a 3-yo, 36 yo, one child) |
| 4.2 “I feel like a hypocrite” | Understanding that their own use and modelling plays a big role in their child’s use | “I’m a bad example because I have one game on my phone that I play once in a while … I know how addictive screens can be and that’s one of the reasons I don’t want my kids started.” (Mother of a 4-yo, 39 yo, 2 children) |
| **Theme 5: Perceived barriers to establishing control over providing mobile screens: The challenges in managing and achieving a balance** | | |
| 5.1 Ideal meets reality | The acceptance that realistically, their imagined ‘ideal’ or ‘perfect’ use of mobile screens is not adherable | “.. this idea of idealism parenting before you have children where you go, “Oh no, I’m not going to let my children play on screens and have access to my phone. Then you get there…, “Holy crap, giving him my phone for five minutes while I am at the doctor’s appointment so he’s quiet and not screaming in my face is actually really handy.” .. going through it has definitely changed my idea of what is okay and what is realistic” (Mother of a 2-yo, 32 yo, 2 children) |
| 5.2 A terrible trade-off | The dilemma parents face due to the challenges in maintaining a balance between costs and benefits to their own wellbeing, of giving screens | “… "Mummy, can you play with me? Mummy, can you do this? Mummy, can you do that?" And just sometimes you have to cook the dinner, ….I feel like sometimes I probably use it more than what maybe I should. But then, sometimes you just have to do what you have to do to get through the day as well.” (Mother of a 2-yo, 38 yo, one child) |
| 5.3 Holding back the tide | The difficulty of being the minority in relation to providing mobile screens | “.. it’s really hard for parents to be able to make an informed choice…a lot of the choice is taken away by the fact that it’s so mainstream now.”(Mother of a 4-yo, 33 yo, 2 children) |
| 5.4 Family matters | Family circumstances pose challenges for maintaining control | “.. if we had another child or something like that, and I was trying to look after a baby and feed a baby and change nappies …, I suspect I'd probably be using it a little bit more. And it really annoys me when my husband is checking his phone, and I feel like saying to him, "You're not hearing a word I'm saying", but then he would say the same thing about me.”(Mother of a 2-yo, 38 yo, only child) |
